# Improved 7 Tesla transmit field homogeneity with reduced electromagnetic power deposition using coupled Tic Tac Toe antennas

**DOI:** 10.1038/s41598-020-79807-9

**Published:** 2021-02-09

**Authors:** Tales Santini, Sossena Wood, Narayanan Krishnamurthy, Tiago Martins, Howard J. Aizenstein, Tamer S. Ibrahim

**Affiliations:** 1 Department of Bioengineering, University of Pittsburgh, Pittsburgh, PA USA; 2 Department of Psychiatry, University of Pittsburgh, Pittsburgh, PA USA; 3 Department of Radiology, University of Pittsburgh, Pittsburgh, PA USA

**Keywords:** Biomedical engineering, Electrical and electronic engineering, Magnetic resonance imaging

## Abstract

Recently cleared by the FDA, 7 Tesla (7 T) MRI is a rapidly growing technology that can provide higher resolution and enhanced contrast in human MRI images. However, the increased operational frequency (~ 297 MHz) hinders its full potential since it causes inhomogeneities in the images and increases the power deposition in the tissues. This work describes the optimization of an innovative radiofrequency (RF) head coil coupled design, named Tic Tac Toe, currently used in large scale human MRI scanning at 7 T; to date, this device was used in more than 1,300 neuro 7 T MRI scans. Electromagnetic simulations of the coil were performed using the finite-difference time-domain method. Numerical optimizations were used to combine the calculated electromagnetic fields produced by these antennas, based on the superposition principle, resulting in homogeneous magnetic field distributions at low levels of power deposition in the tissues. The simulations were validated in-vivo using the Tic Tac Toe RF head coil system on a 7 T MRI scanner.

## Introduction

Magnetic Resonance Imaging (MRI) is excellent for soft tissue imaging and determination of its metabolites. This technology provides high-resolution images with several different contrasts and it is widely utilized in clinical settings. The MRI signal increases with higher static magnetic field strength (B_0_). Therefore, advancing from standard clinical scanners—with B_0_ of 1.5 Tesla (T) or 3 T—to the recent FDA cleared 7 T provides a major advantage of increased signal-to-noise ratio (SNR)^1^. The enhanced SNR can be used either to increase the resolution of the images or to decrease the scanning time (with the use of higher acceleration factors)^1^. Other advantages of 7 T field strength are the higher sensitivity to blood-oxygen-level-dependent (BOLD) signal, better venous vasculature conspicuity, enhanced angiography, and improved spectroscopy acquisitions^1^.

The operational frequency for proton imaging at 7 T is ~ 297.2 MHz. When compared to lower field strength, the shorter wavelengths associated with higher operational frequencies can cause spatial inhomogeneities in the radiofrequency (RF) fields and reduced skin depths; both which can cause voids or regions of low contrast in the images. The higher operational frequencies and RF inhomogeneities can also lead to a higher average and local specific absorption rate (SAR), which can cause temperature rise and potential tissue damage^2^.

Several MRI sequence acquisition methods have been developed to improve the RF excitation homogeneity and insensitivity to inhomogeneities in the circularly polarized component of the RF magnetic field responsible for excitation (B_1_^+^). Some of these methods are adiabatic pulses^3,4^, transmit SENSE^5,6^, spoke pulses^7^, and the acquisition of two interleaved modes with TIAMO^8,9^. This work focuses instead on improving the homogeneity of the B_1_^+^ fields through RF coil design and methodology of operation. Using multichannel transmit RF coil (Tx) systems, the resultant electromagnetic fields can be manipulated with the superposition of the fields generated by each coil element^10^. This technique, known as RF shimming, is accomplished by modifying the phases and amplitudes of the RF field produced by each transmit channel towards specific objectives, usually aiming at increasing the global and/or local B_1_^+^ field homogeneity/intensity and reducing SAR.

Previous works have evaluated the Tic Tac Toe (TTT) RF head coil design for 7 T MRI. In reference^11^, it was demonstrated that a 16-channel TTT multilevel coil can simultaneously drive up to 4 different eigenmodes; one eigenmode from each of the 4 different physical levels of the coil. Reference^12^ provided a theoretical comparison (based on electromagnetic simulations) of the TTT design with the transverse electromagnetic (TEM) resonator, demonstrating an improved transmit field homogeneity and load insensitiveness of the TTT design.

In this work, we describe a methodology for optimizing and operating the TTT transmit coil design for human imaging studies. Constrained numerical optimizations of the 16-channel TTT transmit coil were performed based on finite-difference time-domain (FDTD) electromagnetic field simulations while considering the RF power losses in the hardware. Two homogeneous (in terms of B_1_^+^ field) RF shim cases for two different input power and SAR efficiency levels were experimentally implemented on the single channel (sTx) mode of a MAGNETOM 7 T MRI system using commercially available RF power splitters and phase shifters (coaxial cables). In-vivo B_1_^+^ maps, as well as T2 SPACE (variable flip angle 3D turbo spin echo) and T2 FLAIR (Fluid Attenuated Inversion Recovery) sequences were acquired for demonstration purposes. The results demonstrate homogeneous 7 T neuro imaging. This RF coil system is currently being used in more than 20 patient/disease-based studies funded by NIH (National Institutes of Health). To date, the current fully implemented version of the RF coil system has helped acquire more than 1300 neuro in-vivo scans in ongoing 7T human MRI studies^13,14^.

## Methods

### 16-channel Tic Tac Toe RF coil design

**Figure 1:**
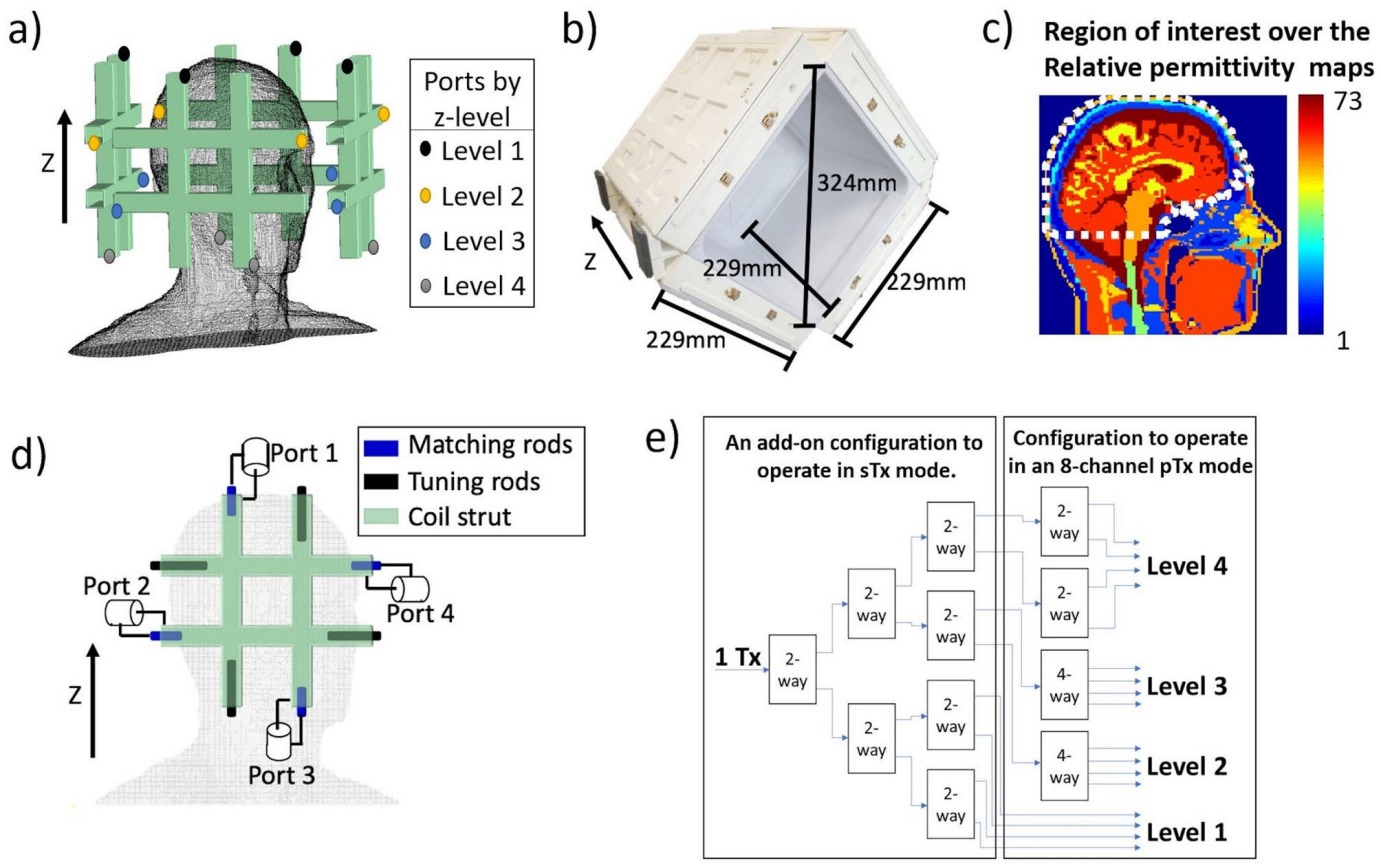
The 16-channel Tic Tac Toe (TTT) Tx head coil FDTD model and experimental implementation. In (**a**), the coil geometry with the locations of the 16 channels of the transmit head coil, which are divided into 4 levels in Z-direction; In (**b**), the assembled 16-channel TTT Tx coil and its dimensions; In (**c**), the region of interest (white dashed line) plotted over the relative permittivity map of the Duke model at ~ 297.2 MHz (7 T proton frequency). In (**d**), the ports, matching rods, and tuning rods of a representative panel of the coil. In (**e**), the RF power splitting configuration—exclusively using 2-way and 4-way Wilkinson power dividers—to drive the 16-channel transmit coil using the system’s sTx or pTx modes. The Tx channels associated with the coil’s levels 1, 2, 3, and 4 (shown in (**a**)) experience normalized voltage amplitudes equal to 1, 0.5, 0.5, and 1/√2, respectively.

The Tx coil is based on the TTT antennas, previously described for foot/ankle^15^, breast^16,17^, and head imaging at 7T^11,12^. Briefly, one TTT panel is composed of eight square-shape transmission lines elements, made using 3D printed polycarbonate and 8 µm-thick copper sheets, connected to each other in a Tic Tac Toe fashion. Four of these elements are connected to excitation ports, and the other four elements are used for frequency tuning. The antennas are matched and tuned by varying the length of the copper rods inside the outer struts (Fig. 1d). No capacitors or inductors are necessary for the matching and tuning of the ports. The Tx coil is composed of four TTT panels positioned around the head, resulting in a 16-channel transmit coil (Fig. 1a,b). The RF shield is composed of double-sided 4 µm-thick copper sheets (Polyflon, Germany). Cuts were added on each side of the copper sheet to reduce eddy currents, as described by Zhao et al.^18^. For optimal imaging purposes, the RF coil system incorporates an in-house developed 32-channel receive (Rx)-only insert^19^.

### FDTD simulations of the Tic Tac Toe coil

The B_1_^+^ and electrical fields were simulated using an in-house developed full-wave FDTD software with an embeded transmission line algorithm for modeling the RF excitation and the coupling^15,17,20–22^. The FDTD solver was developed in C language with multi-thread capability^23^. The spatial resolution utilized for the combined RF coil and load was 1.59 mm isotropic, and the temporal resolution was ~ 3 ps (calculated based on FDTD Courant Condition). This represents a rather fine spatial resolution. For reference, the 10-gm SAR requires averaging more than 2,000 Yee cells. Several works have indicated that a ~ 5 mm isotropic grid would be sufficient for a reasonable accurate modeling^24,25^. The simulation time for one channel to achieve steady state (100,000 time-steps for this model) is about 15 h, allocating 4 cores of an Intel Xeon Gold 6126 processor and about 4 GB of memory. The coil geometry (Fig. 1a) was created using MATLAB (MathWorks, USA), totaling 257 × 257 × 276 or ~ 18 million Yee cells. Perfect matching layers were implemented to absorb the irradiating fields, being 8 layers added on the top of the model (towards the Z direction), 12 on the sides, and 32 on the bottom^26^. Each port/channel was excited individually with a differentiated Gaussian pulse, while all the other ports were terminated with a 50 Ω load using the transmission line numerical model. The Virtual Family Duke model (version 1.0) was used as the load. The model includes the whole head, neck and the top of the shoulders, totaling 23 different tissues. The region of interest (ROI) used includes the head regions from the top of the head through the bottom of the cerebellum and excludes the nasal cavities and ears. The lower ~ 1 cm of the cerebellum volume is excluded as it contains a minimal number of pixels with brain tissues in the Duke model. The contour of the ROI mask applied over the permittivity map is shown in Fig. 1c.

The resolution of the FDTD calculated B_1_^+^ fields was then reduced by a factor of 2 to speed up the RF shimming numerical optimizations (described in the next sections), while the resolution of the electric fields was not changed.

### Strategy for RF shimming

Numerical optimizations designed to minimize specific cost functions were used to manipulate the phases of the RF fields generated by the Tx coil channels. When the optimization goal is to increase B_1_^+^ homogeneity, the coefficient of variation of the B_1_^*+*^ field ($\mathrm{C{V}_{{B}_{1}^{+}}}$), defined as the standard deviation over the mean of the B_1_^+^ inside the ROI, is commonly used as the cost function; however, it may produce local regions of high or low flip angles^27^. Moreover, even if the $\mathrm{C{V}_{{\mathrm{B}}_{1}^{+}}}$ is in its global minimum, SAR levels could be elevated. To overcome these challenges, the cost functions utilized in this work were combinations of $\mathrm{C{V}}_{{\mathrm{B}}_{1}^{+}}$, maximum B_1_^+^ ($\mathrm{max_{{B}_{1}^{+}}}$), minimum B_1_^+^ ($\mathrm{min_{{B}_{1}^{+}}}$), and average SAR. The $\mathrm{C{V}}_{{\mathrm{B}}_{1}^{+}}$ function is continuous and inherently smooth over the multi-dimensional space^28^. As a result, there is a reduced number of local minima produced by this function, which improves the probability of reaching the global minimum for gradient descending algorithms. For this reason, we firstly conducted optimizations using the $\mathrm{C{V}}_{{\mathrm{B}}_{1}^{+}}$ as the cost function and using random phases as the initial conditions (the amplitude values were fixed, following the strategy defined in Fig. 1e). The results from the $\mathrm{C{V}}_{{\mathrm{B}}_{1}^{+}}$ optimizations were then used as the initial conditions for the subsequent optimization, utilizing other cost functions.

The cost functions were minimized using the MATLAB function *fmincon* including GPU acceleration. The algorithm used for the $\mathrm{C{V}}_{{\mathrm{B}}_{1}^{+}}$ optimizations was the active-set, due to its high efficiency and high convergence rate from random initial conditions^29^. This optimization takes about 10 s per run on a NVIDIA Titan RTX GPU in a dual Intel Xeon Gold 6230 system. For the optimizations using other cost functions, the interior-point algorithm was used as it usually performs small steps around the initial conditions instead of larger steps which could affect the results.

There is an inverse relationship between $\mathrm{C{V}}_{{\mathrm{B}}_{1}^{+}}$ and B_1_^+^ efficiency in most multichannel Tx coil designs and a highly homogeneous B_1_^+^ could be achieved at the expense of very low efficiency^30^. In this scenario, a high input voltage is required to achieve the desired flip angle, potentially reaching or exceeding the SAR limits and hardware capabilities. To ensure an adequate efficiency for the Tx coil, the optimizations in this work were performed constraining the mean $\mathrm{{B}_{1}^{+}}$ field to produce 180 degrees flip angle on average with 1 ms square pulse and using 8 kW RF power amplifier. This constraint also considers the measured losses between the RF power amplifier and the RF coil plug (2.72 dB) as well as the losses associated with the coil struts, shields and ports (1.19 dB), coil plug (0.30 dB), phase cables (0.53 dB), and power splitters (0.76 dB).

### RF shimming for the Tic Tac Toe coil

#### Step 1: RF power splitting

The TTT RF coil system was designed to work in either the sTx or parallel-transmit (pTx) modes with the use of Wilkinson power splitters (Fig. 1e). Based on the eigenmodes of the Tx coil design^11^, the 16-channel Tx coil is composed of 4 excitation levels spatially positioned in the Z direction (Fig. 1a); each level is composed of 4 Tx coil channels. In this work, Level 1 was chosen to be excited with half of the total supplied RF power because of the higher power efficiency associated with this level and its capacity to produce center bright, as previously demonstrated in reference^11^. Levels 2 and 3 play an essential role in exciting the lower brain regions, including the cerebellum and the temporal lobes^11^. One-eighth of the total supplied RF power was used to excite each of these two levels. Level 4, which produces a similar B_1_^+^ pattern of Level 1^11^, received one-quarter of the total supplied RF power. In order to compare this arrangement with other possible configurations, RF shimming optimizations were performed by randomly permutating the Tx coil channels amplitudes with the four possible values of amplitudes, as described in Fig. 1e, and then performing phase-only optimizations using the cost function and steps shown in Fig. 3c.

#### Step 2: Optimization of the B_1_^+^ field and SAR for a specific RF power splitting configuration

SAR calculation was incorporated into the optimization software by sampling the electric fields (a voxel was randomly sampled every 4 × 4 × 4 voxels) and calculating the average SAR in these samples. This method represents a good estimation of the global average SAR and was used to speed up the optimizations. It is worth noting that this method was only applied inside the iterations of the optimization software, the final SAR maps were calculated by averaging the original—high resolution—electric fields (without sampling) for every 10 g of tissue, including partial volume calculation as described in previous work^31^. The objective function in Eq. (1) was minimized in multiple optimizations, aiming at achieving a compromise between B_1_^+^ homogeneity and SAR reduction. Each optimization—including the post hoc SAR calculation—takes approximately 3 min using the cost function described in Eq. 1 with a NVIDIA Titan RTX GPU in a dual Intel Xeon Gold 6230 system.

1 $$\mathrm{cost=\frac{C{V}_{{B}_{1}^{+}}}{0.17}+x\times\frac{{Ma{x}_{{B}_{1}^{+}}}/{Mi{n}_{{B}_{1}^{+}}}}{3}+y\times\frac{SAR}{1.5}}$$

where $\mathrm{C{V}_{{B}_{1}^{+}}}$, $\mathrm{Ma{x}_{{B}_{1}^{+}}}$, $\mathrm{Mi{n}_{{B}_{1}^{+}}}$ are, respectively, the coefficient of variation, the maximum, and the minimum of the $\mathrm{{B}_{1}^{+}}$ fields inside the region of interest; the constants 0.17, 3, and 1.5 are roughly the expected optimal values of the $\mathrm{C{V}_{{B}_{1}^{+}}}$, $\mathrm{{Ma{x}_{{B}_{1}^{+}}}/{Mi{n}_{{B}_{1}^{+}}}}$, and SAR, respectively. Dividing the $\mathrm{C{V}_{{B}_{1}^{+}}}$, $\mathrm{{Ma{x}_{{B}_{1}^{+}}}/{Mi{n}_{{B}_{1}^{+}}}}$, and SAR by the expected values was used to normalize the data and avoid bias, since the resultant value is approximately equal to one for each term of the cost function; the unit of $\mathrm{SAR}$ is W/Kg for an average $\mathrm{{B}_{1}^{+}}$ of 2μT in the ROI; the values of x and y randomly variate in every optimization in order to change the weights of $\mathrm{{Ma{x}_{{B}_{1}^{+}}}/{Mi{n}_{{B}_{1}^{+}}}}$ and SAR, respectively, inside the cost function.

### Experimental implementation

Two RF shim cases were experimentally implemented using coaxial cables as fixed phase shifters and Wilkinson power splitters (Fig. 1e). The imaging experiments - all done on the sTx mode - were conducted in a whole body 7 T scanner (MAGNETOM, SIEMENS, Germany) with 8 kW power amplifier capabilities. In-vivo images were acquired in healthy volunteers with informed consent as part of an approved study by the University of Pittsburgh’s Institutional Review Board (identification number PRO17030036). All procedures complied with relevant guidelines and regulations for investigational use of the device in humans.

B_1_^+^ maps were acquired using the Turbo-FLASH sequence^32^ with the following parameters: TR/TE = 2000/1.16 ms; flip angle from 0° to 90° in 18 degrees increments; acquisition time = 12 min, resolution 3.2 mm isotropic. The output was fitted to a cosine function to produce the flip angle maps. For demonstration purposes, 2D FLAIR and 3D T2-SPACE sequences were used to acquire whole-brain images. The respective sequences parameters were: (1) 2D FLAIR, TE/TI/TR = 103/2,900/13,500 ms, BW = 230 Hz/pixel, resolution 0.7 × 0.7 × 2 mm^3^, acceleration factor 2, 4 interleaved acquisitions (64 transversal slices), total acquisition time = 7:36 min; (2) 3D T2-SPACE sequence (WIP692), TE/TR = 369/3400 ms, BW 488 Hz/pixel, T1/T2 = 1500/250 ms, acceleration factor 3, with 224 slices in transversal acquisition, acquisition time = 8:11 min, and bias corrected using the SPM12 package^33^.

## Results

**Figure 2:**
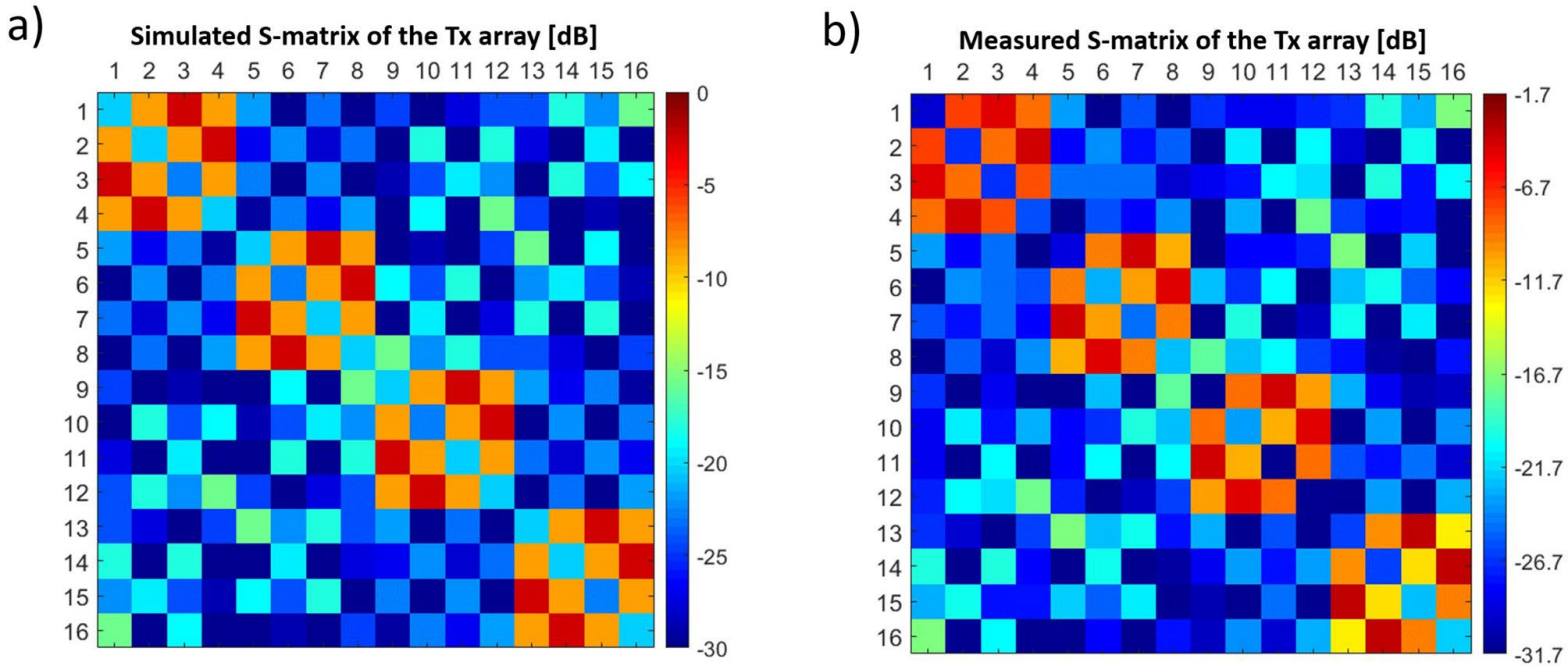
S-parameter comparison between simulations and experiments of the Tic Tac Toe 16-channel Tx head coil. In (**a**), the FDTD simulated s-matrix using a transmission line model mechanism; in (**b**), the experimentally measured s-matrix of the constructed head coil. The color scale limits were modified to compensate for the electrical losses in the constructed coil (not included in FDTD model).

Figure 2a and b compares the measured and simulated scattering parameters of the 16-channel TTT Tx coil. The simulated and measured values have Pearson correlation coefficient of 0.935. The measured coupling between the opposite ports of each TTT panel (highest coupling of the design) was − 4.26 dB on an average while the simulated value was − 2.68 dB on average. This difference is attributed to electrical losses in the copper, elements, and ports of the Tx coil, which are not included in the FDTD model. The measured maximum and average coupling between any pair of panels were − 17.0 dB and − 24.4 dB, respectively.

**Figure 3:**
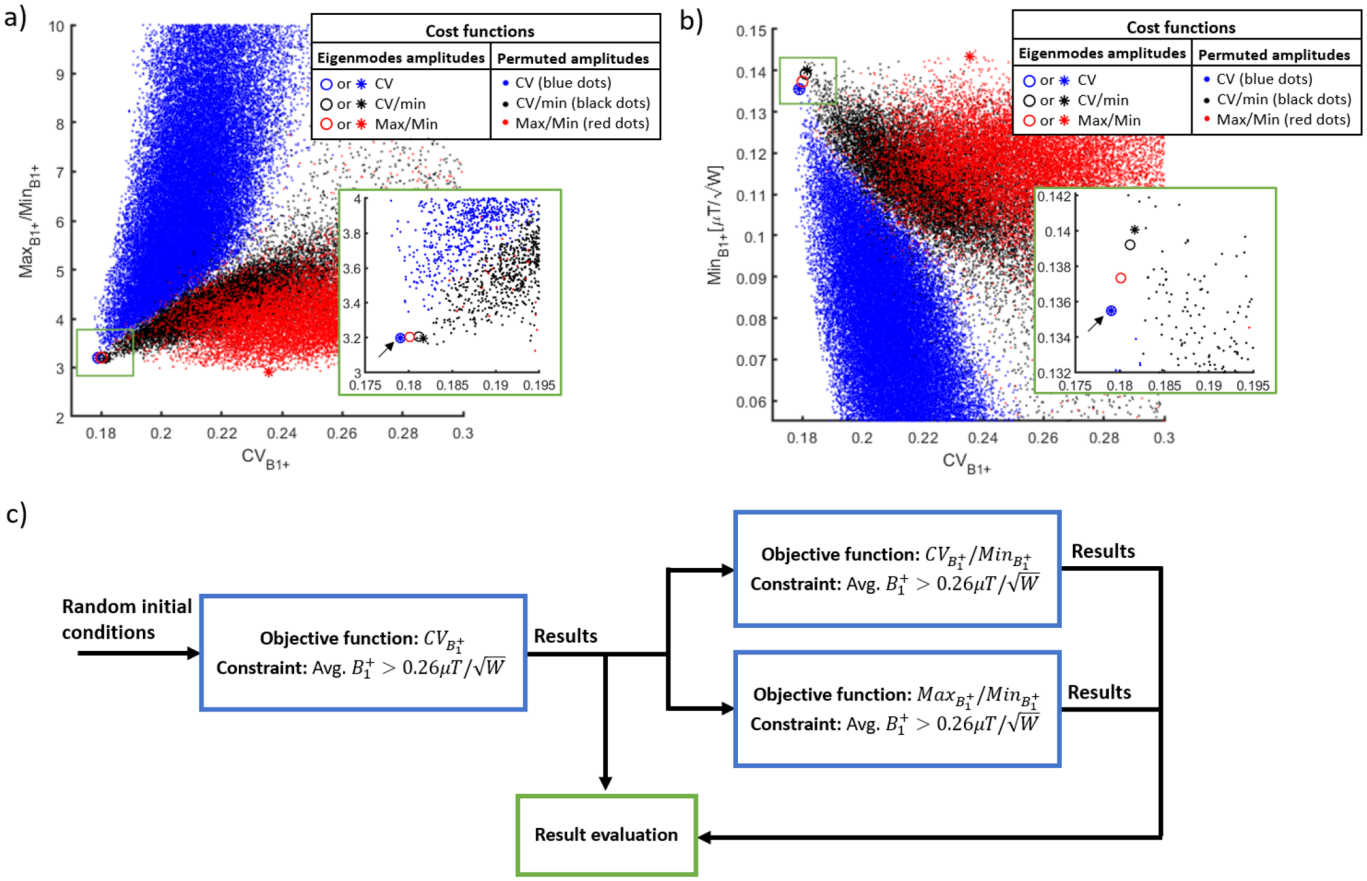
Phase-only B_1_^+^ RF shim cases for the 16-channel Tic Tac Toe RF coil. This analysis investigates the performance of several configurations using 2-way and 4-way splitters for implementation on the sTx mode. In (**a**, **b**), the phase-only RF shim cases, with the amplitude scheme derived from the eigenmodes of the RF coil (described in Fig. 1b) and in reference^11^, were compared with RF shimming optimizations where the amplitudes of the Tx channels were randomly permutated but can only take on normalized values = 1, 1/√2, or 0.5. Approximately 300,000 optimizations were performed, presented as the colored dots. The cost functions for the RF shimming optimizations were the ${\mathrm{CV}}_{{\mathrm{B}}_{1}^{+}}$, ${\mathrm{CV}}_{{\mathrm{B}}_{1}^{+}}/{\mathrm{min}}_{{\mathrm{B}}_{1}^{+}}$ , and ${\mathrm{max}}_{{\mathrm{B}}_{1}^{+}}/{\mathrm{min}}_{{\mathrm{B}}_{1}^{+}}$, following the flowchart in (**c**). The region of interest for the B_1_^+^ field stats is the entire head from cerebellum excluding the nasal cavities and the ears (Fig. 1c). The black arrows point to the case selected as initial condition for the next optimizations, which was chosen due to having a combination of low ${\mathrm{CV}}_{{\mathrm{B}}_{1}^{+}}$, low ${\mathrm{max}}_{{\mathrm{B}}_{1}^{+}}/{\mathrm{min}}_{{\mathrm{B}}_{1}^{+}}$, and high ${\mathrm{min}}_{{\mathrm{B}}_{1}^{+}}$. The circles represent the RF shim cases with the best ${\mathrm{CV}}_{{\mathrm{B}}_{1}^{+}}$ and the asterisks are the cases with the best ${\mathrm{max}}_{{\mathrm{B}}_{1}^{+}}/{\mathrm{min}}_{{\mathrm{B}}_{1}^{+}}$ for each cost function.

Figure 3a and b shows the effect of permutating the values of the amplitudes associated with each individual Tx channel on the overall B_1_^+^ performance. The RF power splitting scheme shown in Fig. 1e is compared with approximately 300,000 permutations of the amplitude values followed by phase-only RF shimming. The results—utilizing the RF power splitting scheme from the eigenmodes of the RF coil (Fig. 1e)—demonstrate superior performance in terms of $\mathrm{C{V}}_{{\mathrm{B}}_{1}^{+}}$, $\mathrm{{max}}_{{\mathrm{B}}_{1}^{+}}$/$\mathrm{{min}}_{{\mathrm{B}}_{1}^{+}}$, and $\mathrm{{min}}_{{\mathrm{B}}_{1}^{+}}$. The RF shim case indicated by the black arrow in Fig. 3a and b was chosen as the starting point for the next step in the optimizations, utilizing the cost function shown in Eq. (1).

**Figure 4:**
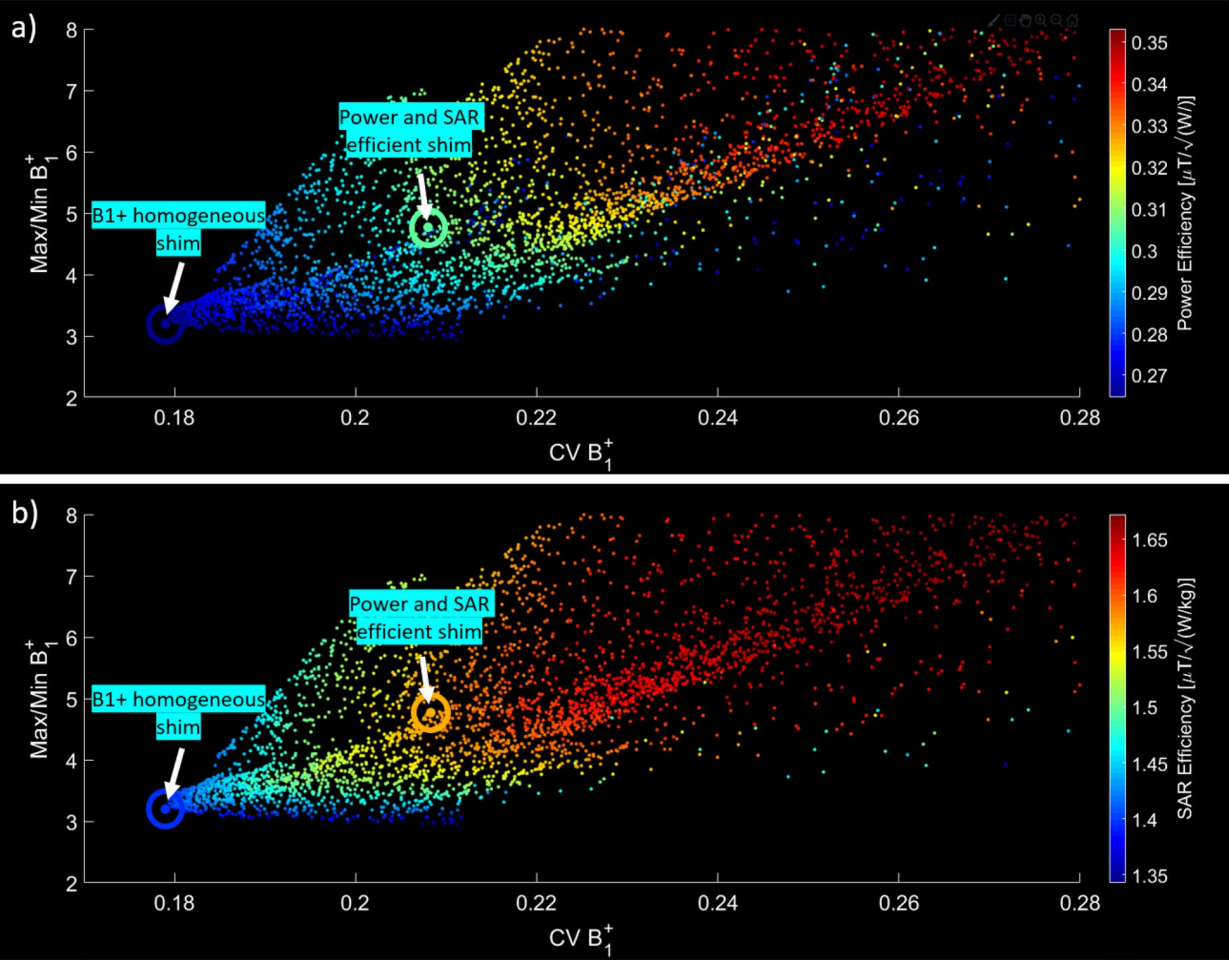
SAR and B_1_^+^ phase-only RF shimming of the 16-channel Tic Tac Toe RF coil. The B_1_^+^ homogeneity parameters (${\mathrm{CV}}_{{\mathrm{B}}_{1}^{+}}$ and ${\mathrm{max}}_{{\mathrm{B}}_{1}^{+}}/{\mathrm{min}}_{{\mathrm{B}}_{1}^{+}}$ in X and Y axes of the plot, respectively) are compared with the power efficiency (**a**) and SAR efficiency (**b**). Each point corresponds to an RF shim case using a cost function (Eq. 1) that includes ${\mathrm{CV}}_{{\mathrm{B}}_{1}^{+}}$, ${\mathrm{max}}_{{\mathrm{B}}_{1}^{+}}/{\mathrm{min}}_{{\mathrm{B}}_{1}^{+}}$, and average SAR. Average B_1_^+^ constraints were also included. Two RF shim cases were chosen for experimental implementation: (1) power and SAR efficient shim and (2) B_1_^+^ homogeneous shim.

Figure 4a and b shows the characteristics of the B_1_^+^ field and SAR variating the weights of the cost function (Eq. 1) and variating constraints on the average B_1_^+^ intensity. While higher levels of SAR efficiency (i.e., lower average SAR) and higher power efficiency can be achieved, it usually comes at the cost of lower levels of B_1_^+^ field homogeneity (higher $\mathrm{C{V}_{{\mathrm{B}}_{1}^{+}}}$ and/or $\mathrm{{max}}_{{\mathrm{B}}_{1}^{+}}$/$\mathrm{{min}}_{{\mathrm{B}}_{1}^{+}}$). From the scattering plots, we chose two cases for experimental implementation (arrows in Fig. 4a,b): “B_1_^+^ homogeneous shim” situated in the edge of the graph, representing the maximum homogeneity in the lower limit of power efficiency, and “Power and SAR efficiency shim” which is situated in the middle of the graph and represents a compromise between B_1_^+^ homogeneity, B_1_^+^ efficiency, and SAR efficiency.

**Figure 5:**
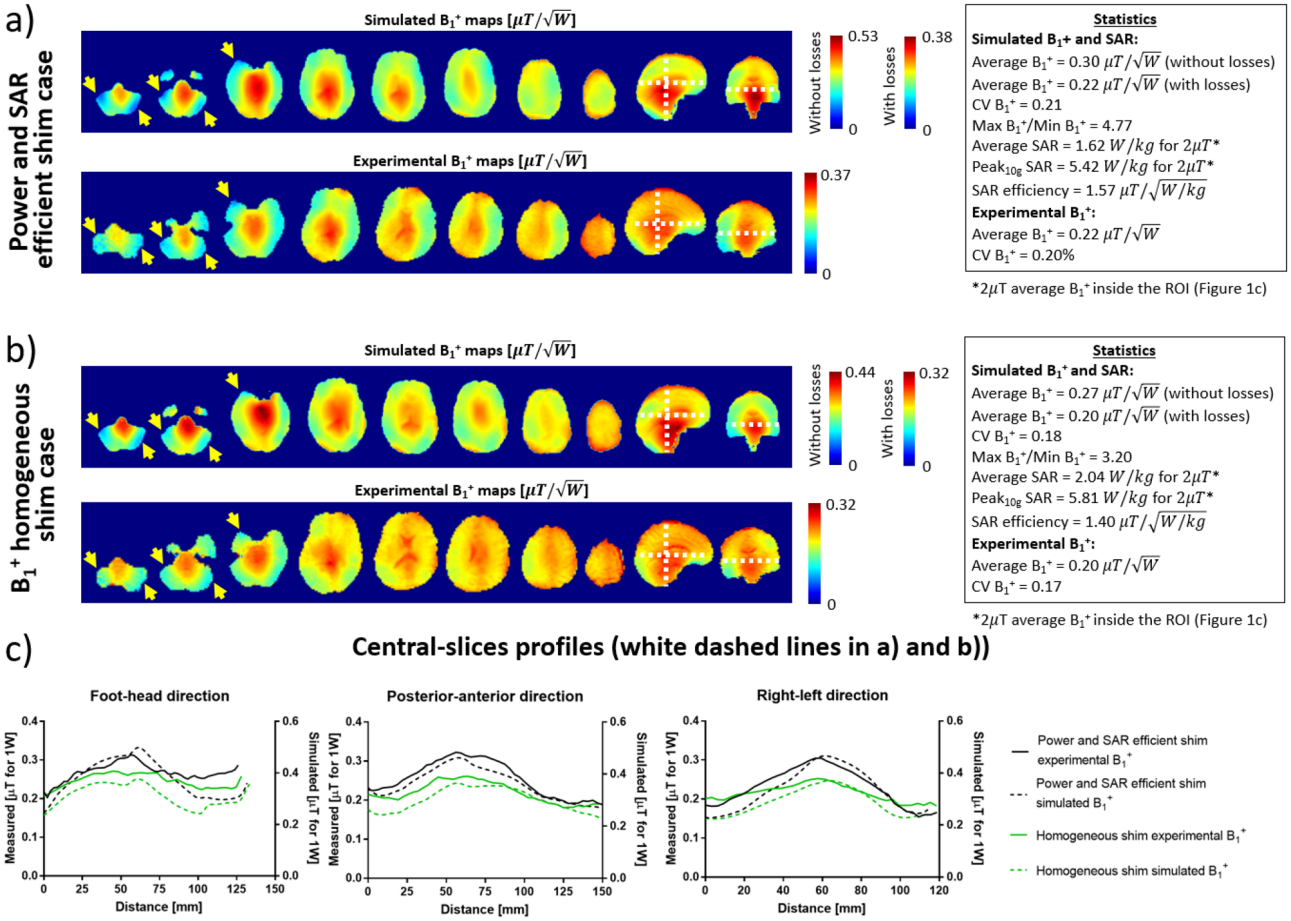
Simulated and experimental B_1_^+^ maps comparisons for 2 RF shim cases. In (**a**), simulated and in-vivo B_1_^+^ maps and statistics of the power and SAR efficient RF shim case. In (**b**), simulated and in-vivo B_1_^+^ maps for the B_1_^+^ homogeneous RF shim case. The statistics from the simulations were calculated over the region of interest shown in Fig. 1c. The yellow arrows point to regions of low B_1_^+^ that were mitigated with the B_1_^+^ homogeneous RF shim case. In (**c**), the central slice profiles from the simulated and experimental B_1_^+^ maps [white dashed lines in (**a**) and (**b**)]. The differences observed in the mean B_1_^+^ are due to the electrical losses in the coil structure/cables/plugs/ports/connections/splitters. With a linear system and superposition principle, the overall loss is included as part of the simulation data (“With losses” colorbar). Since the overall electrical loss does not affect B_1_^+^ distribution (represents a fixed drop in the intensity), the calculated CV values for the simulations and experiments are comparable for the two RF shim cases (20% vs. 21% and 17% vs. 18%). After including the overall measured electrical loss (27.4% in voltage as described in “Methods” section), the maximum and average values for the simulations and experiments are also comparable, 0.38 vs. 0.37 and 0.32 vs. 0.32 $\mathrm{\mu T}/\sqrt{\mathrm{W}}$ for maximum B_1_^+^ field, and 0.22 vs. 0.22 and 0.20 vs. 0.20 $\mathrm{\mu T}/\sqrt{\mathrm{W}}$ for the average B_1_^+^ field.

**Figure 6:**
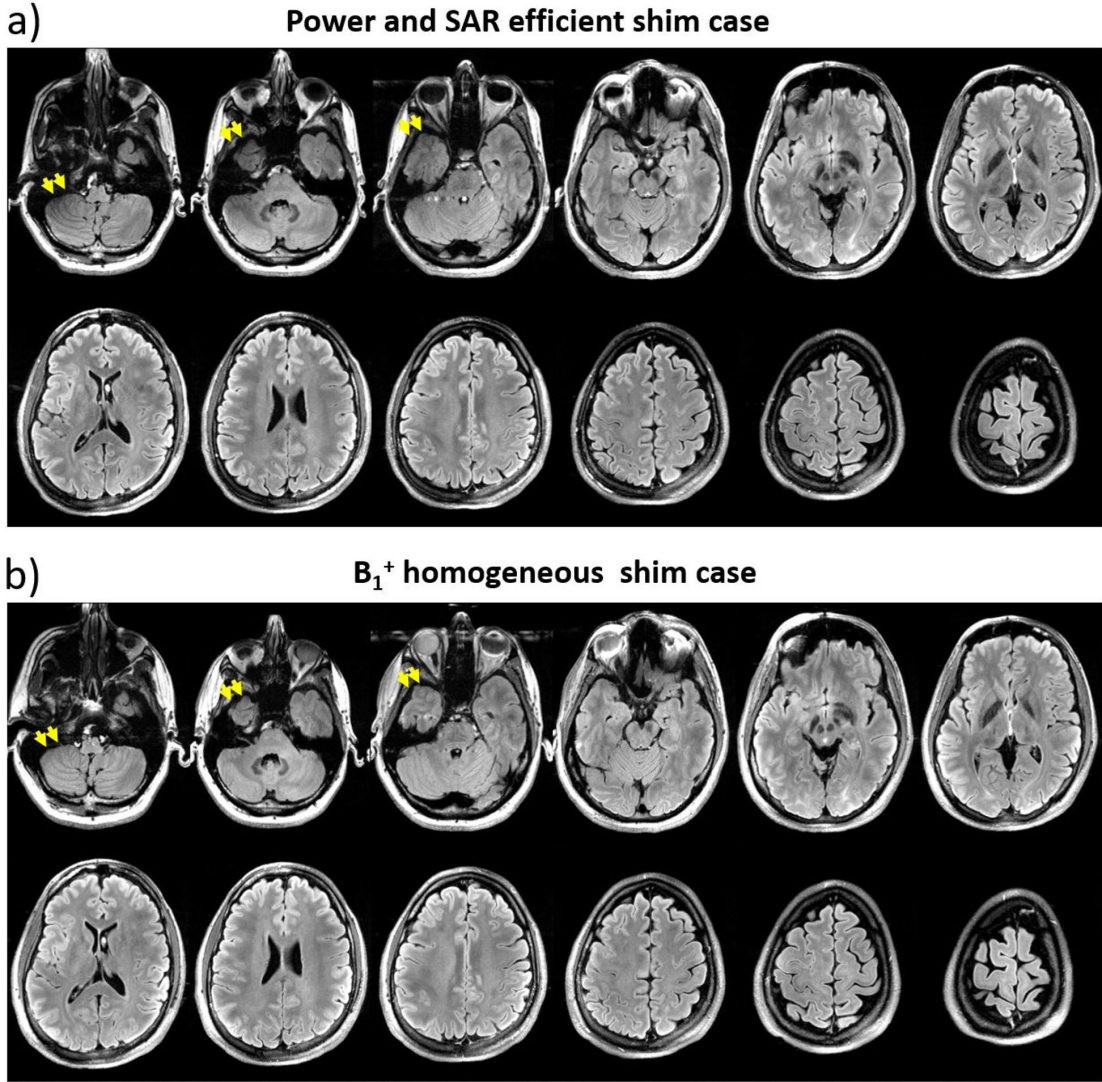
Axial slices of the FLAIR images acquired at resolution of 0.7 × 0.7 × 2 mm^3^. The images show the differences between the power and SAR efficient (**a**) and B_1_^+^ homogeneous (**b**) shim cases. The yellow arrows point to the regions where the dropout was mitigated by the B_1_^+^ homogeneous case. The parameters of the acquisition were: TE/TI/TR = 103/2900/13,500 ms, acceleration factor 2, BW = 230 Hz/Px, transversal acquisition of 64 slices, field of view 224 × 176.4 mm^2^ in axial plane, and acquisition time = 7:36 min.

Fig. 5a–c shows a comparison of the two experimentally implemented, non-subject-specific RF shim cases. Specifically, the comparison shows the experimental B_1_^+^ field maps and the corresponding simulated results on the Duke model. In Fig. 5a, a power and SAR efficient RF shim case is shown. It achieves a SAR efficiency of 1.57 $\mathrm{\mu T/\sqrt{W/kg}}$ (simulated), average B_1_^+^ of 0.22 $\mathrm{\mu T/\sqrt{W}}$ (experimental), $\mathrm{C{V}}_{{\mathrm{B}}_{1}^{+}}$ of 20% (experimental), and $\mathrm{{max}}_{{\mathrm{B}}_{1}^{+}}$/$\mathrm{{min}}_{{\mathrm{B}}_{1}^{+}}$ of 4.77 (simulated). The B_1_^+^ homogeneous RF shim case (Fig. 5b) has a SAR efficiency of 1.40 $\mathrm{\mu T/\sqrt{W/kg}}$ (simulated), average B_1_^+^ of 0.20 $\mathrm{\mu T/\sqrt{W}}$ (experimental), $\mathrm{C{V}}_{{\mathrm{B}}_{1}^{+}}$ of 17% (experimental), and $\mathrm{{max}}_{{\mathrm{B}}_{1}^{+}}$/$\mathrm{{min}}_{{\mathrm{B}}_{1}^{+}}$ of 3.20 (simulated). Figure 5c shows the profiles in the central slices of the experimental and simulated B_1_^+^ field for the two RF shim cases. The impact of having lower B_1_^+^ field intensities can be seen near the yellow arrows displayed over the B_1_^+^ field maps in Fig. 5a and b. Figure 6 shows the effects of the RF shimming on the FLAIR images, acquired on the same volunteer.

**Figure 7:**
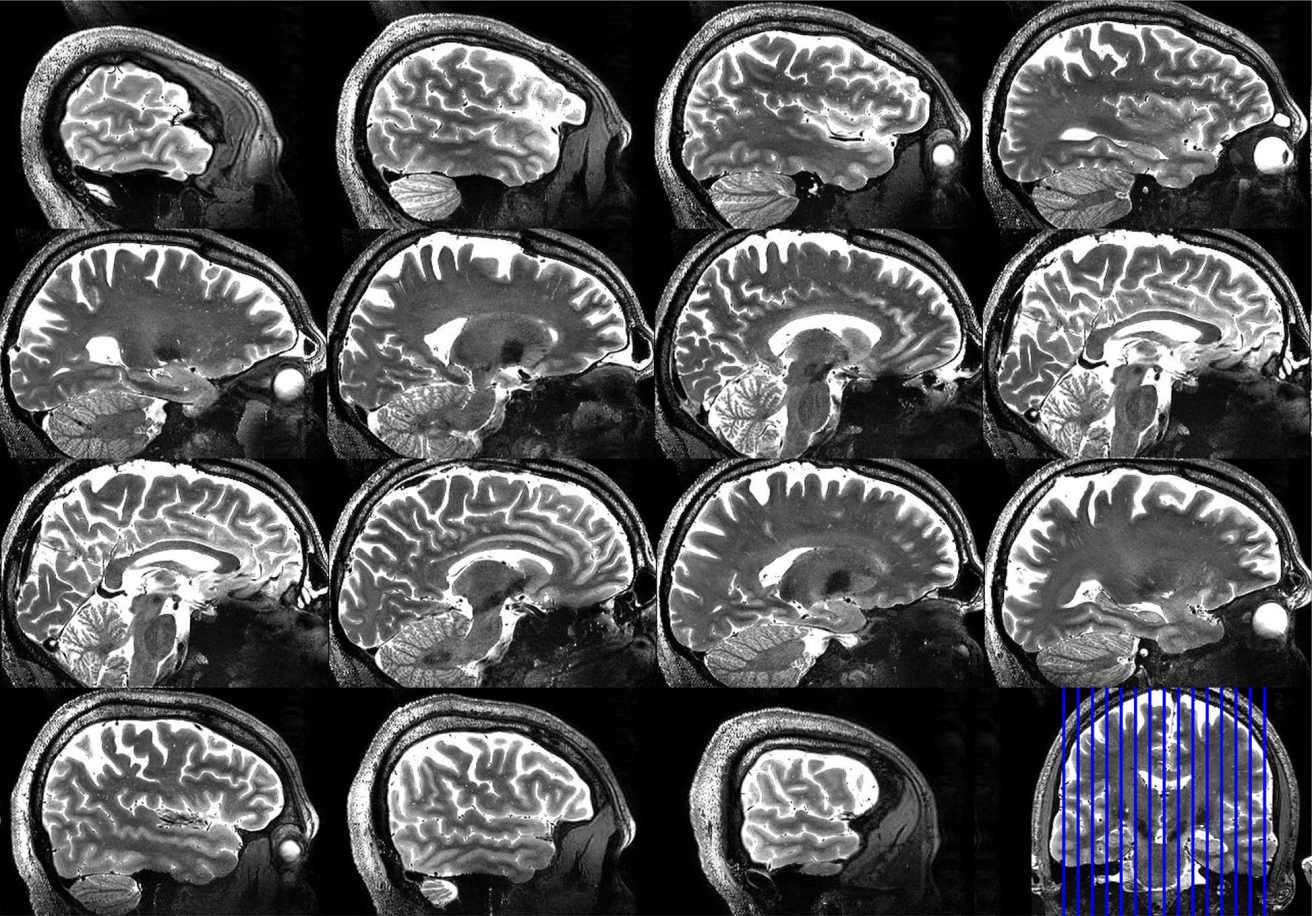
Sagittal slices of the 3D-SPACE acquired at 0.6 mm isotropic resolution, showing full brain and cerebellum coverage in a volunteer with large head size (~ 205 mm in anterior–posterior direction measured from the forehead). The images were obtained using the B_1_^+^ homogeneous RF shim case on the sTx mode. The parameters of the acquisition were: TE/TR = 369/3400 ms, acceleration factor 3, BW = 488 Hz/Px, transversal acquisition of 224 slices, field of view 192 × 165.6 mm^2^ in axial plane, and acquisition time = 8:11 min.

Figure 7 shows representative slices of the T2-weighted 3D SPACE images acquired in a volunteer with large head size (approximately 205 mm in anterior–posterior direction from the forehead) using the B_1_^+^ homogeneous RF shim case shown in Fig. 5b. Using an MR sequence that requires high levels of B_1_^+^ homogeneity, the images show that the implemented RF shim case provides non-subject specific homogeneous field distribution and it achieves full brain coverage – including the cerebellum and temporal lobes – in a relatively larger head.

## Discussions

In this work, a 16-channel transmit coil based on the Tic Tac Toe design was optimized using non-subject specific phase-only RF shimming. The RF shimming approach combined with the coil’s 4 excitation levels in the Z direction can significantly impact the Tx coil performance by introducing tradeoffs among RF power efficiency, B_1_^+^ field homogeneity, and SAR. These parameters can be controlled, depending on the imaging application and the RF amplifier capacity, by changing the lengths of the coaxial cables feeding the coil or by using pTx systems.

### S-matrix comparison

The RF TTT transmit coil was simulated with a transmission line algorithm for modeling the excitation and coupling as part of the in-house developed FDTD package^23^. Accurate electromagnetic modeling of the coil’s coupling is critical for implementing RF shimming fully based on B_1_^+^ and electric fields calculated from electromagnetic simulations. The simulated and measured s-matrices are comparable (Fig. 2a,b, respectively), with a Pearson correlation coefficient of 0.935. There is an offset in the values (− 1.7 dB on average) that is related to the losses in the coil and connectors. We did not include the losses in the simulations since we believe there is still a margin for improvement. For example, using less-porous CNC-machined Teflon parts instead of 3D printed polycarbonate may reduce the losses in the coil.

### Strategy for power splitting among the channels

Previous work^11^ has showed that the 16-channel RF coil contains different levels of power efficiencies in accordance with the position of the ports in Z direction (direction of the static magnetic field). The voltage amplitudes for the Tx channels were chosen so that the most efficient coil levels are excited with a larger fraction of the supplied RF power. This approach produces improved B_1_^+^ homogeneity (Fig. 3a) and high minimum B_1_^+^ intensity inside the ROI (Fig. 3b) when compared with other possible combinations using the same RF power splitter configuration. All cases presented can be easily implemented using 2-way and 4-way RF power splitters and coaxial cables as phase shifters on the system’s sTx or pTx modes.

### B_1_^+^ and SAR RF shimming

Figure 4a and b shows the flexibility of the phase-only RF shimming scheme in combination with the 16-channel TTT coil design, demonstrating the tradeoff among SAR efficiency, B_1_^+^ efficiency, and B_1_^+^ homogeneity. Based on the electromagnetic simulations, a homogeneity of 18% ($\mathrm{C{V}}_{{\mathrm{B}}_{1}^{+}}$) can be achieved within the ROI, resulting in a SAR efficiency of 1.40 $\mathrm{\mu T/\sqrt{W/kg}}$ and an average B_1_^+^ of 0.27 $\mathrm{\mu T}$. Increasing the weight of the SAR in the cost function (Eq. 1) and increasing the mean B_1_^+^ constraint improved the SAR efficiency to 1.57 $\mathrm{\mu T/\sqrt{W/kg}}$ while maintaining a $\mathrm{C{V}}_{{\mathrm{B}}_{1}^{+}}$ of 21% and an average B_1_^+^ field of 0.30 $\mathrm{\mu T}$. These values of SAR efficiency and B_1_^+^ field homogeneity represent an improved performance for 7 T RF head coils when compared with traditional RF coils. As a comparison, Krishnamurthy et al.^12^ reported the values of SAR efficiency of 1.27 $\mathrm{\mu T/\sqrt{W/kg}}$ and homogeneity of 27% for the TEM resonator, using the same simulation environment utilized in this work. Other studies investigated the SAR performance for several other coil designs^34–37^, however the simulations were performed in different software environments and often using different head models, which makes the comparison with this work challenging.

In general, the cost function combining $\mathrm{C{V}_{{\mathrm{B}}_{1}^{+}}}$, $\mathrm{{max}_{{\mathrm{B}}_{1}^{+}}}$/$\mathrm{{min}_{{\mathrm{B}}_{1}^{+}}}$, and $\mathrm{SAR}$ with the average $\mathrm{{B}_{1}^{+}}$ constraint presented a good performance for this particular RF coil design, as it allowed different operational points where it is possible to minimize $\mathrm{C{V}_{{\mathrm{B}}_{1}^{+}}}$, $\mathrm{{max}_{{\mathrm{B}}_{1}^{+}}}$/$\mathrm{{min}_{{\mathrm{B}}_{1}^{+}}}$, or maximize SAR efficiency (Fig. 4a,b), depending on the application. It is important to note that the optimal cost function may vary depending on the RF coil design, the constraints included in the simulation, and the ROI used.

### Experimental verification

Figure 5a and b shows the experimental implementation of the two RF shim cases and the corresponding simulated B_1_^+^ maps. The first case is intended for higher SAR and RF power efficiency; the second case is optimized for higher B_1_^+^ homogeneity. The images—using a high flip angle sequence (FLAIR)—shown in Fig. 6 demonstrate the differences between the two RF shim cases in the cerebellum and temporal lobe, which are very challenging regions at 7 T MRI^38,39^. Although the B_1_^+^ homogeneous RF shim case presents lower RF power efficiency, the improved B_1_^+^ homogeneity justify its use, since the lower RF power efficiency can be compensated by adjusting the input voltage amplitude or by increasing the pulse width of the sequences.

Figure 5c shows a comparison between the simulated and experimental B_1_^+^ profiles. The differences in the intensity can be explained by the losses in the splitters/cables/connectors (approximately 17% measured loss in voltage) and losses in the coil. The differences in the field distribution can be attributed to the model approximations and differences between the head model size/position and the in-vivo. For instance, the brain in the model used is about 14 cm long in foot-head direction, while the volunteer is about 12.5 cm.

The 3D T2-SPACE whole-brain and cerebellum images, shown in Fig. 7, were acquired at 7 T using the B_1_^+^ homogeneous RF shim case implemented on the sTx mode (Fig. 5b). The results demonstrate that homogeneous whole-brain imaging in a volunteer with a large head size is achievable with a challenging T2-weighted sequence at 7 T MRI^40–42^—often associated with significantly lower signal in the temporal lobe and cerebellum.

The RF coil design presented in this work has lower power efficiency, but better SAR efficiency, when compared to more traditional 7 T RF head coil designs. The lower power efficiency is attributed to the strong coupling between opposite elements of the TTT coil, which causes a significant portion of the transmitted power to be dissipated in the RF power splitters and the scanner system’s circulator. For instance, the two RF shim cases presented in this work have a mean B_1_^+^ of 0.27 and 0.30 $\mathrm{\mu T/\sqrt{W}}$ in the simulations (not considering losses in the hardware). Using the same software environment, Krishnamurthy et al.^12^ demonstrated that the 4-channel, 16-element TEM resonator (with the same length as the 16-channel TTT coil) presents a power efficiency of 0.45 $\mathrm{\mu T/\sqrt{W}}$. However, considering all the power losses in the system and in the coil, the 16-channel TTT coil still provides enough mean B_1_^+^ intensity to have inversion using 1 ms square pulse with 8 kW power amplifier (standard in older 7 T scanners), which is sufficient for most imaging applications. The B_1_^+^ homogeneous RF shim case (Fig. 5b) is currently being heavily used with the sTx mode on more than 20 NIH patient/disease studies conducted in our facility^13,14^ due to its high B_1_^+^ homogeneity and extended coverage in challenging-to-image regions in the head at 7 T.

## Data Availability

The data that support the findings of this study are available from the corresponding author upon request.
